# Photometric Calibration for Stereo Camera with Gamma-like Response Function in Direct Visual Odometry

**DOI:** 10.3390/s21217048

**Published:** 2021-10-24

**Authors:** Yinming Miao, Masahiro Yamaguchi

**Affiliations:** 1 Interdisciplinary Graduate School of Science and Engineering, Tokyo Institute of Technology, Yokohama 226-8503, Kanagawa, Japan; 2 School of Science and Engineering, Tokyo Institute of Technology, Yokohama 226-8503, Kanagawa, Japan; yamaguchi.m.aa@m.titech.ac.jp

**Keywords:** direct visual odometry, stereo camera, vignetting, response function, stereo matching

## Abstract

Direct visual odometry algorithms assume that every frame from the camera has the same photometric characteristics. However, the cameras with auto exposure are widely used outdoors as the environment often changes. The vignetting also affects the pixel’s brightness on different frames, even if the exposure time is fixed. We propose an online vignetting correction and exposure time estimation method for stereo direct visual odometry algorithms. Our method works on a camera that has a gamma-like response function. The inverse vignetting function and exposure time ratio between neighboring frames are estimated. Stereo matching is used to select correspondences between the left image and right image in the same frame at the initialization step. Feature points are used to pick the correspondences between different frames. Our method provides static correction results during the experiments on datasets and a stereo camera.

## 1. Introduction

With the fast development of digital cameras, more and more computer vision systems are applied in various fields, such as visual navigation robotics. Visual odometry algorithms have been improved greatly. Direct methods, such as LSD-SLAM (Large-scale direct monocular SLAM) [1], DSO (Direct Sparse Odometry) [2], LDSO (Direct Sparse Odometry with Loop Closure) [3], Stereo DSO [4], and SO-DSO (Scale Optimized Direct Sparse Odometry) [5], have been proposed. These methods assume that the same point in different frames appears same pixel value. However, for auto exposure cameras, which could adapt to different light environments, the observed pixel value from the same scene point may change during a time in the sequence. This affects the precision of the direct visual odometry algorithms.

Worse yet, there is an irradiance fall-off phenomenon called vignetting. The irradiance reduces on the periphery compared to the image center. It means that, even the exposure time is fixed, the pixel value changes if the camera is moving and the projected location of the scene point to the camera is changing.

Prior photometric camera calibration has been proved effective for improving the performance of DSO. Therefore, it is necessary to align the brightness of the same scene point in different frames. For this purpose, the vignetting phenomenon is required to be estimated. Furthermore, the relationship between the irradiance captured by the camera and the output image brightness value is often nonlinear, which makes the estimation more complex. The camera response function (CRF) is used to describe the relationship between the irradiance and the image brightness.

There are also other color correction factors, such as white balance correction, for multi-camera systems. In most visual odometry systems, only grayscale images or one channel of the color images are used for processing. Thus, we just discuss the grayscale images in this work.

In some cases, the response function and vignetting function are estimated beforehand. However, to achieve the response function in this case, the camera and the scene objects should remain static, and the environment light source should be fixed. In fact, if the exposure time and other parameters change during recording, it is more desirable to use the captured images to estimate the response function [6]. For the vignetting function, a uniform radiance plane is usually used as a given reference target [7], and the function that describes the relationship of pixel brightness and location is established. However, sometimes, there is no condition to do the pre-calibration, such as the exposure time could not be set manually. In addition, the change of aperture or focus affect the vignetting, the calibrated function under fixed settings may not be suited to different actual operations.

There are also many calibration methods for the captured images without special patterns. In some multi-view systems and image-stitching tasks, the overlap area gives many correspondences, so that we could analyze the brightness change rule of one object point in different views or images. A. Litvinov et al. [8] propose a linear least squares solution for response and vignetting functions, where they use the correspondences obtained from panorama stitching. In the visual odometry system, the camera usually moves at fast forward speed. Drastic changes in perspective relationships affect the performance of the image stitching method. S. J. Kim et al. [9] propose a method to estimate the radiometric response function of the camera and the exposure difference between frames with feature tracking. M. Grundmant et al. [10] suppose the camera response function changes with scene content and exposure and propose a self-calibration model for time-varying mixture of responses. In these methods, they suppose that the movement in the track is short, and no vignetting is present. In order to improve the performance of monocular direct visual odometry, P. Bergmann et al. [11] propose an energy function for response and vignetting at the same time. The correspondences for the function are captured from the KLT tracker. In direct visual odometry with stereo cameras [4], the exposure differences between neighboring frames are estimated without considering the vignetting effects.

In this paper, we propose a new calibration method to establish the vignetting function and exposure time ratios between frames from a stereo camera used in direct visual odometry. Feature points tracking and stereo matching give out the correspondences for solving the energy function. The method is suitable for the camera with a gamma-like response function. We calibrate the captured images with the inverse vignetting function and the exposure time ratios during the visual odometry processing. We tested our algorithm on open datasets and image sequences captured by a stereo camera.

## 2. Method

### 2.1. Photometric Model

In this paper, we suppose that every object in the scene has a Lambertian surface. The radiance of a Lambertian surface to a camera is the same regardless of the camera’s angle of view. Although there may be some objects that do not follow Lambertian reflectance, if we can get enough points pairs on the Lambertian surface, the solving processing of energy equation will be robust. When *L* is the radiance of a point in the scene, *t* is the exposure time of the camera, and then the irradiance of the point at the sensor should be tL in the ideal situation. However, the vignetting effect appears in most camera systems. V(x) is used to describe the vignetting effect of the camera, and *x* is the location of the point in the image sensor. The real irradiance of one point in the sensor is V(x)tL.

In order to make the image taken by a camera to be adapted to the color space standard, digital camera normally uses a nonlinear response function to transform the irradiance on the sensor to image pixel brightness value. However, the response function in fact depends on the camera devices to generate visually preferable images. The function *f* describes the CRF. For an 8-bit image, which is normally used in image processing, the range of *f* is [0,255]. Here, we normalize the range to [0,1] for convenience. The brightness value of a scene point in the image captured by the camera can be written as

I=f(tV(x)L).

For the point $p^{i}$ in *k*-th frame with exposure time $t_{k}$, the value in the image can be written as

$$I_{k}^{i}=f\left(t_{k}V\left(x_{k}^{i}\right)L_{i}\right).$$

Here, $L_{i}$ is a fixed value for the object corresponding to the pixel $p^{i}$ based on the Lambertian reflection assumption. We can get the function:

$$\frac{f^{-1}\left(I_{k}^{i}\right)}{V\left(x_{k}^{i}\right)t_{k}}=L_{i}.$$

It is easy to think of an energy equation in the below format:

$$E=\sum_{k=1}^{n}\sum_{i\in P}{\left(\frac{f^{-1}\left(I_{k}^{i}\right)}{V\left(x_{k}^{i}\right)t_{k}}-L_{i}\right)}^{2}.$$

Here, *n* is the totally used frame number and *P* is the set of tracked points. As the $L_{i}$ is unknown, we change the format of energy equation to

$$E=\sum_{k=1}^{n-1}\sum_{i\in P}{\left(\frac{f^{-1}\left(I_{k+1}^{i}\right)}{V(x_{k+1}^{i})}-\frac{f^{-1}\left(I_{k}^{i}\right)t_{k+1}}{V\left(x_{k}^{i}\right)t_{k}}\right)}^{2}.$$

We cannot know the real exposure time but only the ratio of exposure time between frames.

### 2.2. Vignetting Function Model

In our method, a polynomial is used to approximate the vignetting correction function.

$$\tilde{V}\left(x\right)=\frac{1}{V(x)}=1+\sum_{i=1}^{n}v_{i}R{\left(x\right)}^{i}.$$

Here, R(x) is the normalized radius of the image point to the optical center of the image. If the optical center is not known, the center of the image is used. In most SLAM datasets or stereo camera systems, the optical center is necessary for odometry-related processing. We could consider the location of the optical center as known information.

### 2.3. Response Function Model

Grossberg and Nayar [12] introduced a diverse database of real-world camera response functions (DoRF). They combine the basis functions from DoRF to create a low-parameter Empirical Model of Response (EMoR), such as:

$$f\left(x\right)=g_{0}\left(x\right)+\sum_{s=1}^{n}c_{s}h_{s}\left(x\right).$$

Here, $g_{0}\left(x\right)$ is the mean response function, and $c_{s}$ is the parameter for each basic function $h_{s}$. Many camera manufacturers design the response to be a gamma curve. Therefore, they included a few gamma curves, chosen from the range 0.2≤γ≤2.8, in the database.

Then, we consider the camera with a CRF in the type of

$$f\left(l\right)=l^{\gamma },$$

where *l* is the normalized irradiance, l∈[0,1], γ>0. We get f(0)=0, f(1)=1, and *f* is a monotonically increasing function.

We assume that there is an ideal solution with a response function $f_{0}\left(l\right)=l^{\gamma_{0}}$, a vignetting function $V_{0}$, and the exposure time of *k*-th frame is $t_{k}^{0}$. With this solution, we get

$$\frac{f_{0}^{-1}\left(I_{k+1}^{i}\right)}{V_{0}\left(x_{k+1}^{i}\right)}=\frac{f_{0}^{-1}\left(I_{k}^{i}\right)t_{k+1}^{0}}{V_{0}\left(x_{k}^{i}\right)t_{k}^{0}}.$$

Then, for another $\gamma_{j}$, which is a given constant different from $\gamma_{0}$, we can give out another solution:

(1) $$\left\{\begin{array}{c} f_{j}\left(l\right)={\left(f_{0}\left(l\right)\right)}^{\gamma_{j}/\gamma_{0}}=l^{\gamma_{j}},\\ V_{j}\left(x\right)={\left(V_{0}\left(x\right)\right)}^{\gamma_{0}/\gamma_{j}},\\ t_{k}^{j}={\left(t_{k}^{0}\right)}^{\gamma_{0}/\gamma_{j}}. \end{array}\right.$$

With this solution, we can also get

$$\frac{f_{j}^{-1}\left(I_{k+1}^{i}\right)}{V_{j}\left(x_{k+1}^{i}\right)}={\left(\frac{f_{0}^{-1}\left(I_{k+1}^{i}\right)}{V_{0}\left(x_{k+1}^{i}\right)}\right)}^{\gamma_{0}/\gamma_{j}}={\left(\frac{f_{0}^{-1}\left(I_{k}^{i}\right)t_{k+1}^{0}}{V_{0}\left(x_{k}^{i}\right)t_{k}^{0}}\right)}^{\gamma_{0}/\gamma_{j}}=\frac{f_{j}^{-1}\left(I_{k}^{i}\right)t_{k+1}^{j}}{V_{j}\left(x_{k}^{i}\right)t_{k}^{j}}.$$

Thus, there are infinite solutions for the equation without extra assumption.

We also use several other kinds of functions to estimate the response function, such as polynomial, power function (gamma curve) with constant term and functions in Reference [12]. We tested these methods on different datasets, and they all give an unstable performance.

### 2.4. Assumptions

Usually, the left camera and right camera in a stereo camera system have the same light-sensitive components and the same type of lens. Some stereo camera systems have hardware synchronization that makes the exposure time settings for the multi-cameras exactly the same. Even without hardware synchronization, the same auto-exposure algorithm and highly overlapping fields of view make the exposure time of the left camera and the right camera almost the same in most conditions. In our method, the response functions of the left camera and right camera are assumed to be same. Even though each lens has a slight difference, we still can assume that the vignetting effects are the same.

Gamma correction is a widely used nonlinear operation for encoding and decoding luminance or tristimulus values in video or still image systems for compensating the display tone reproduction curve [13]. The image sensors used in the industrial application usually have a gamma correction parameter in settings. Some cameras for image processing have a linear CRF, which means a gamma curve with γ=1. Thus, in our method, we assume the CRF is described with a gamma curve. In the previous section, we discuss that, if there is a ideal solution for the energy equation, for any γ, we can get another solution. Especially, we set the CRF function as f(x)=x. Although we cannot get the real solution, we can use the special solution to unify the pixels in different frames which are corresponding to the same object into the same brightness value.

**Figure 1:**
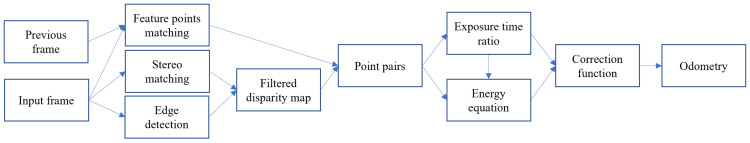
Flow chart of the process of the proposed method.

Figure 1 shows the flow chart of the whole processing.

### 2.5. Stereo Matching

**Figure 2:**
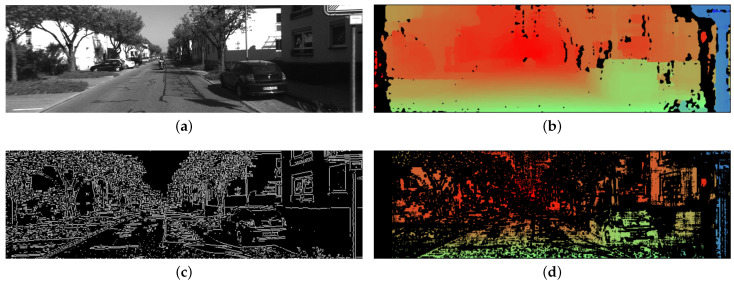
The disparity image is transformed from grey image to color image for visualization. The value of disparity is normalized and used as hue channel value of HSV ((Hue, Saturation, Value) color space. The pixel which is black means the disparity is unknown. The edge image is captured from the left image. The white pixels show the edge area. (**a**) Image from KITTI dataset [16] sequence 00 (left camera). (**b**) Disparity image. (**c**) Edge image. (**d**) Disparity image applied filter.

Stereo matching algorithms have been greatly developed in recent years. We use stereo matching between image pairs in the initialization step of the algorithm to get a quick estimate of the vignetting function. Here, we focus on the computational cost. The accuracy of each point and density of the disparity image are not much concerned as we do not use the disparity to calculate the depth. The semi-global matching [14] method is chosen to process the stereo matching. This is a mature algorithm, which can be realized on GPU. The internal and external parameters of stereo camera are necessary before stereo matching, where they are often provided in a visual odometry system. So, it is reasonable to assume that those parameters are already known in the processing. During the stereo matching, each point gets a cost value to measure the confidence of the matching. Usually, the pixel with high confidence should be chosen. In stereo matching, the edge points or corner points often get a higher confidence value. The disparity is a sub-pixel value, and the brightness changes a lot on the edge area. It is not easy to achieve exact correspondences of brightness on the edge area. As the applied method is a dense matching, we can get enough correspondences even if the points on the edge area are not selected. In order to move out the pixels on the edge area from the data used for estimation, the canny detector [15] is applied to extract the edge. Then, we apply a blur kernel to the edge image. The pixel value of the blurred image is used to measure the distance of the pixel to the nearby edge. Finally, we select pixels with high confidence and far away from edges to get correspondences. Figure 2 shows a sample of a filtered disparity image.

In the algorithm, we resize the input image to speed up the stereo matching. Then, an interpolation method is processed to resize the disparity map to its original size. We also give out a GPU accelerated version of the stereo matching program to ensure the processing in real-time.

### 2.6. Feature Points Mapping

We choose the ORB-features [17] to process feature points mapping between frames. The ORB-feature is robust to rotation and illumination change, so it is suitable for our target. We calculate the feature points mapping between the previous frame and the current frame. Further, we calculate the feature points mapping between the left and right frames in the same frame pair. As we assume the left and right frames have the same exposure time, the difference between the same point in the two frames occurred by the vignetting effects. One point may have different projected coordinates in the left camera and right camera because of the baseline of the two cameras. Thus, the radius of the point to the optical center changes in the left camera and right camera. These two frames from one frame pair give more stable information for vignetting estimation than two frames that have different exposure times.

To estimate the exposure time ratio between two frames, it is easy to consider using the matched feature points and calculating the ratio of their pixel brightness. However, as the camera often keeps moving during the visual odometry processing, the location of one point in the frame changes during time. The vignetting affects the pixel value in each frame. If we just use the matched points pair in two frames with different radius without an inverse vignetting function, the precision will be affected. In the early step of the estimation, vignetting function is unknown. It is a challenge for a monocular camera algorithm to fix this problem. In stereo camera system, we could use the previous left camera to match the current camera and also use the previous right camera to match the current left camera. Much more feature points pairs could be achieved. We give a threshold for the difference of radius of the two points in one pair. Only when the points have almost the same radius will the points pair be used in the estimation. In addition, a weight coefficient inversely proportional to the radius is used to reduce the vignetting effect.

After the estimation of the exposure time ratio, we give out a threshold to decide whether use the previous frame pair to achieve more points pairs for vignetting function or not. If the exposure time rate is very close to 1, we assume that the camera did not change the exposure setting between the two frames. The feature points pairs between the previous frame pair and current frame pair are used to achieve more information for vignetting estimation. The brightness values of neighboring pixels of points pairs are used to increase the precision. We stop this step when enough points have been selected for the calculation of the vignetting function. The next exposure time estimation is the same as the original direct visual odometry. In this paper, we apply our method on Stereo DSO and SO-DSO.

### 2.7. Energy Equation

We assume that the CRF is f(x)=x, and the energy equation is simplified as follows:

$$E=\sum_{k=1}^{n-1}\sum_{i\in P}{\left(\frac{I_{k+1}^{i}}{V(x_{k+1}^{i})}-\frac{I_{k}^{i}t_{k+1}}{V\left(x_{k}^{i}\right)t_{k}}\right)}^{2}.$$

$r_{k}=t_{k+1}/t_{k}$ is calculated by the points pairs with similar radius to optical center before solving the equation. Then, we change the energy equation format to

$$E=\sum_{k=1}^{n-1}\sum_{i\in P}{\left(I_{k+1}^{i}\tilde{V}\left(x_{k+1}^{i}\right)-I_{k}^{i}r_{k}\tilde{V}\left(x_{k}^{i}\right)\right)}^{2}.$$

Here, $\tilde{V}$ is the vignetting correction function which is unknown in the equation. We use SVD decomposition to solve this least square problem.

## 3. Evaluation

### 3.1. KITTI Dataset

The KITTI dataset [16] is a well-known odometry benchmark. Stereo images and ground truth are provided in several sequences captured in the urban environment. However, the exposure time of each frame is unknown in this dataset. The left and right cameras have almost the same exposure time. We use the point pairs from left and right images which have similar radius to the optical center to estimate the exposure time ratio between the left and right images.

**Figure 3:**
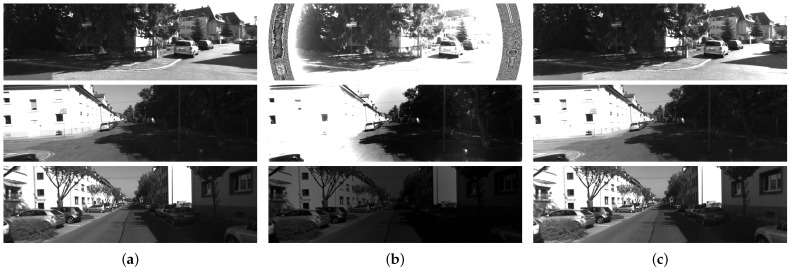
The experimental results of KITTI dataset 00 sequences. (**a**) The original images of KITTI 00 sequences. (**b**) Corrected images by Reference [11]. (**c**) Corrected images by our method.

**Figure 4:**
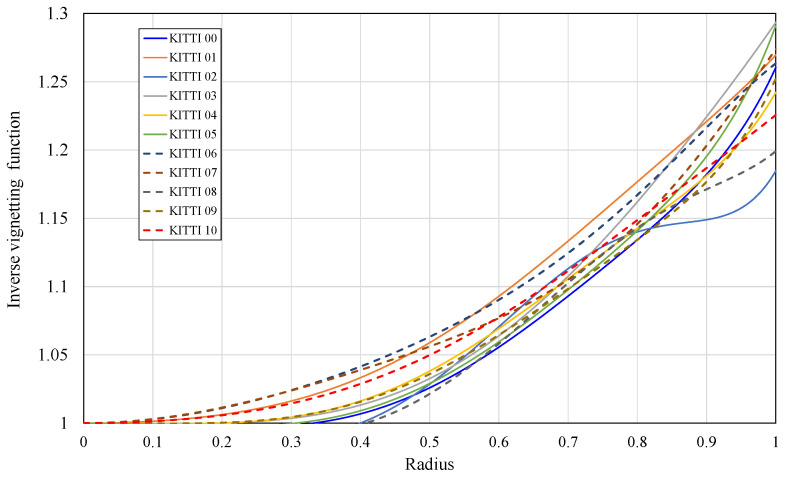
Inverse vignetting function versus radius of sequences 00 to 10 in KITTI dataset.

First, we test the algorithm in Reference [11] with its open-source implementation. We take the left camera view as the target input in the algorithm. The default parameters in the open source implementation are used in the experiment. We also apply our algorithm on the same dataset but using both left view and right view. Although we cannot compare the evaluated exposure time with the real exposure time, the corrected images of Reference [11] show an obviously failed result in Figure 3. Figure 4 shows the estimated inverse vignetting functions on sequence 00 to 10 in KITTI dataset.

As the main purpose of the paper is to improve the accuracy of visual odometry, we test the Stereo DSO and SO-DSO combined with our method. There is no official open source implementation of Stereo DSO, so we develop the algorithm based on DSO open-source implementation. There are many parameters in the implementation, and our results of Stereo DSO are not as good as the results in the paper [4]. However, we can still compare the results of our implementation with and without the proposed method.

We apply Stereo DSO and SO-DSO on the first 11 sequences in the datasets. The results in Table 1 show that our method achieves a better result than Stereo DSO. However, the improvement is not very significant. The vignetting usually affects the pixels on the corners of a square image. In the KITTI dataset, the images are narrow rectangles. Thus, the pixels affected by the strong vignetting phenomenon may be already cut off. In some sequences, such as #00, #04, the improvement is confirmed. Table 2 contains the results on SO-DSO and shows a similar situation as in Table 1.

The experiments are carried out on a computer with an Intel i7-6700 CPU and an NVIDIA GeForce GTX 1050 GPU. The GPU is used to accelerate depth map calculation. The initialization step of our method is time consuming, and the sequences in the KITTI dataset have different numbers of frames. Therefore, the average time per frame of each sequence will change greatly. We compare the time of the two stages separately. In the process of calculating the inverse vignetting function, the additional average time per frame, excluding the odometry processing, is 309.747 ms. After the initialization step, we apply the inverse vignetting function from the beginning of the sequences to compare the time costs of corrected images and original images on odometry algorithms. Here, we do not compare the time cost of Stereo DSO and SO-DSO but compare their effects with and without our method, respectively. Table 3 shows that Stereo DSO and SO-DSO work faster on corrected images in most sequences.

### 3.2. OpenLORIS-Scene Dataset

**Figure 5:**
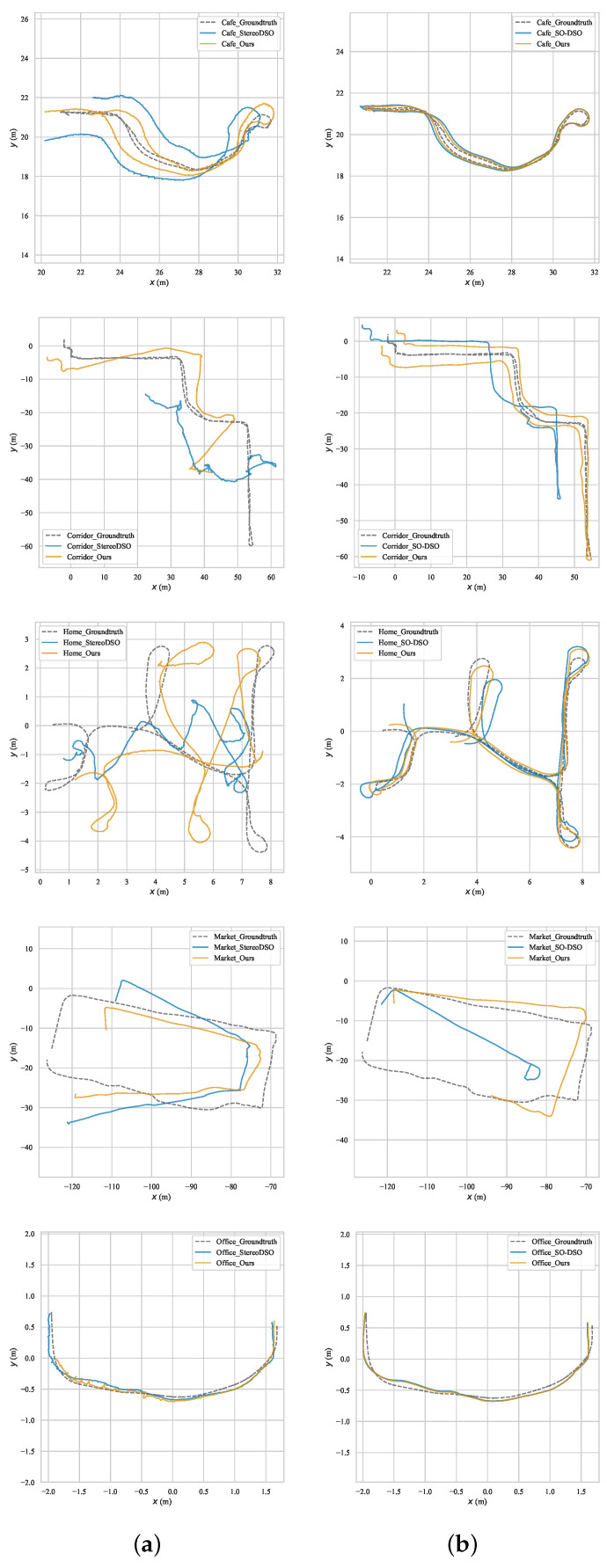
The experimental results on OpenLORIS-Scene dataset. (**a**) Trajectories by Stereo DSO with and without the proposed method. (**b**) Trajectories by SO-DSO with and without the proposed method.

The OpenLORIS-Scene dataset [18] contains sequences from 5 scenes. The stereo fisheye images in the dataset are captured by RealSense T265 [19]. The vignetting phenomenon is obvious in the fisheye images. Both Stereo DSO and SO-DSO faced challenges in this dataset. We applied the proposed method with Stereo DSO and SO-DSO. Figure 5 shows the trajectories of the first sequences of each scene on the OpenLORIS-Scene dataset. All the calculated trajectories performed Sim(3) alignment to the ground-truth. The results show that the trajectories by our method are more similar to the ground-truth, especially more than Stereo DSO.

### 3.3. Stereo Camera

For demonstrating more the effectiveness of the proposed method, we choose a stereo camera LeadSense N1 [20], which is produced by Shanghai Eyevolution Technology Co., Ltd., Shanghai, China. This camera could give out real exposure time. The default CRF of this camera is f(x)=x. The two cameras are synchronized, and the focal lengths are fixed. We set the auto exposure mode to region of interest (ROI) mode. In this mode, the camera selects part of the image to adjust the exposure time. We set the ROI to a 100 × 100 rectangle on the top left corner of the image. When the camera is moving, the scene usually changes fast on the corner of the image. Thus, the exposure time changes faster then normal condition. The resolution of the image is 1280×720 pixels. The camera is held by human hand and moves with strong rotation around an office.

**Figure 6:**
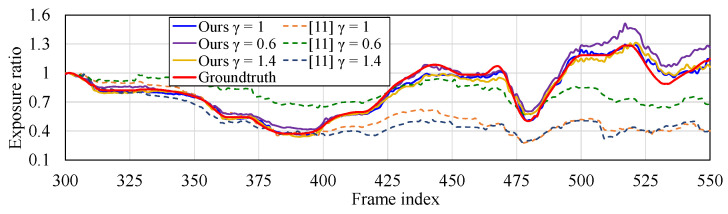
Ground truth of exposure time from a sequence capture by a stereo camera. Evaluated exposure times by Reference [11] and our method are compared with the ground truth.

We test three γ of gamma-curves to the images from the camera. We compare the exposure time rate of current frame and previous frame calculated by our method and Reference [11] with ground truth. The exposure ratio by our method in Figure 6 is calculated as follows:

$$ratio_{k}={\left(r_{k}\right)}^{1/\gamma },$$

where *k* is the frame index.

As shown in Figure 4, the estimated exposure ratio is significantly closer to the true value than the comparison method in all γ values. This makes the exposure calibration between frames feasible.

**Figure 7:**
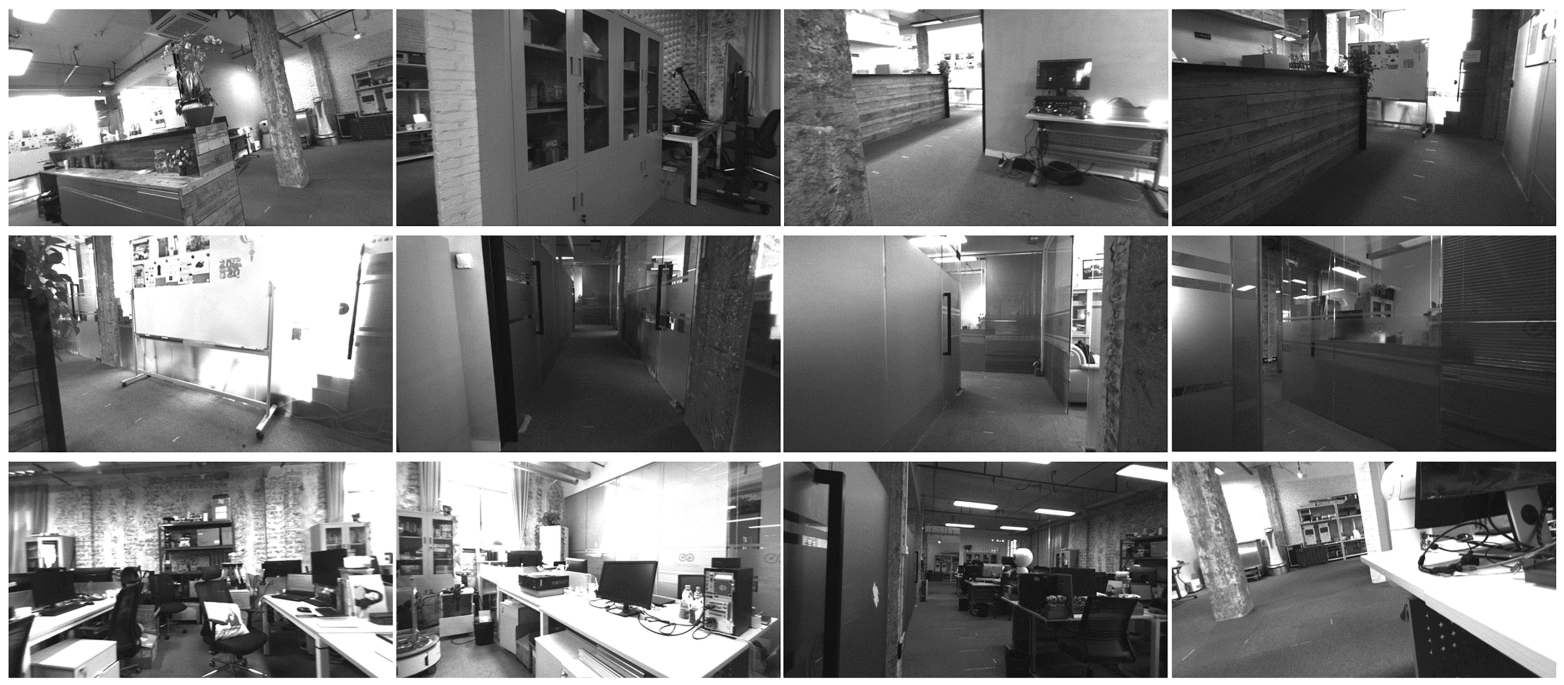
Samples of the sequence captured in an office.

We also compare the reconstructed map by our method and stereo DSO. The camera is also held by human hand and makes a circle around the office. Samples of the captured images in the office are shown in Figure 7. The office is about 200 square meters, and the sequence contains 1312 frames.

**Figure 8:**
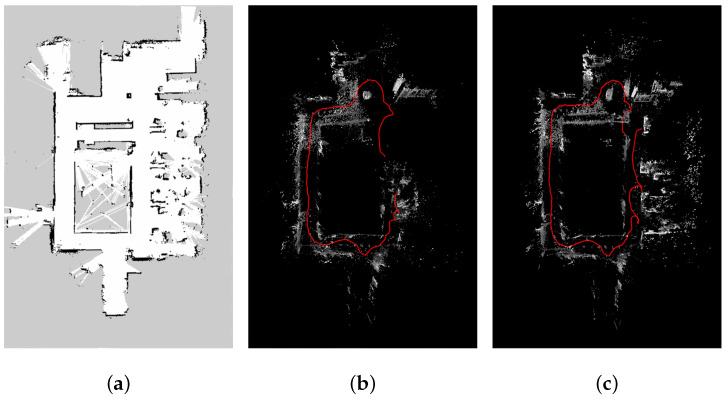
The visual odometry results of a sequence captured by a stereo camera. (**a**) The reconstructed map of an office by laser radar. (**b**) The reconstructed map by Reference [11]. (**c**) The reconstructed map by our method.

As we have no ground truth of the camera movement or the map, we use a laser radar YDLIDAR G4 [21] on a remote control car TurtleBot2 [22] to create a map for reference. The reconstructed maps in Figure 8 are captured from top-view. The red trajectories are the calculated camera trajectories. The result of our method shows more complete structure of the office and is more similar to the result captured by the radar.

## 4. Conclusions

We present a vignetting and exposure time estimation method for a stereo camera in a visual SLAM system. The method is suitable for the camera with a gamma-like response function. In the beginning stage of processing, the disparity map is used to get corresponding points between the left image and right image in one frame pair. The parallel computing in GPU reduces the processing time of the disparity map generation step. The exposure time rate between frames is calculated from the brightness of feature point pairs between frames combined with vignetting reverse function. The method works when the left and right cameras are synchronous or have similar exposure time. The experiments are applied on an open dataset and our stereo camera. The results of the experiment on the open datasets show that our method improves the accuracy of odometry, though the vignetting phenomenon is not obvious in this dataset. At the same time, the existing photometric calibration method shows its limitations in some sequences. We also apply the proposed method to our stereo camera data and show that the proposed method outperforms the conventional calibration method in the exposure time rate estimation. The odometry results are visually compared with the map reconstructed from the laser radar data, and the effectiveness of the proposed method is confirmed.

## Figures and Tables

**Table 1:** Results of Stereo DSO with and without the proposed method on the KITTI dataset. $t_{rel}$ translational RMSE (%), $r_{rel}$ rotational RMSE (degree per 100 m). Best results are shown in bold type.

	Stereo-DSO with Our Method		Stereo DSO	
**Seq.**	$t_{\mathrm{rel}}$	$r_{\mathrm{rel}}$	$t_{\mathrm{rel}}$	$r_{\mathrm{rel}}$
00	**0.9871**	**0.0036**	0.9908	0.0069
01	**1.9327**	**0.0007**	1.9602	0.0007
02	**0.7835**	**0.0024**	0.7846	0.0024
03	0.9860	0.0020	**0.9578**	**0.0020**
04	**0.8795**	**0.0015**	1.0487	0.0023
05	**0.9307**	**0.0036**	0.9352	0.0036
06	0.7235	0.0032	**0.7085**	**0.0032**
07	0.9848	0.0052	**0.9307**	**0.0047**
08	**1.2247**	**0.0034**	1.2307	0.0034
09	**1.0573**	**0.0020**	1.0602	0.0020
10	**0.4934**	**0.0019**	0.4949	0.0019
mean	**0.9985**	**0.0027**	1.0093	0.0030

**Table 2:** Results of SO-DSO with and without the proposed method on the KITTI dataset. $t_{rel}$ translational RMSE (%), $r_{rel}$ rotational RMSE (degree per 100 m). Best results are shown in bold type.

	Stereo-DSO with Our Method		Stereo DSO	
**Seq.**	$t_{\mathrm{rel}}$	$r_{\mathrm{rel}}$	$t_{\mathrm{rel}}$	$r_{\mathrm{rel}}$
00	**1.9137**	**0.0053**	1.9430	0.0054
01	2.4935	**0.0021**	**2.4876**	0.0023
02	**1.5320**	**0.0042**	1.5790	0.0044
03	3.3254	0.0039	**3.1587**	**0.0039**
04	**1.9332**	**0.0025**	1.9928	0.0026
05	**1.7524**	**0.0019**	2.0662	0.0040
06	**2.0650**	**0.0041**	2.2160	0.0051
07	2.9020	0.0061	**2.7461**	**0.0060**
08	**2.0257**	**0.0044**	2.0827	0.0044
09	2.2449	**0.0046**	**2.2295**	0.0047
10	**1.4299**	**0.0045**	1.4343	0.0046
mean	**2.1471**	**0.0040**	2.1760	0.0043

**Table 3:** Time costs of Stereo DSO and SO-DSO on the original KITTI dataset and the corrected images by the proposed method. Best results are shown in bold type.

	Stereo DSO		SO-DSO	
**Seq.**	**Original** **(ms)**	**Corrected** **(ms)**	**Original** **(ms)**	**Corrected** **(ms)**
00	72.807	**49.086**	96.875	**96.149**
01	**49.235**	54.025	**68.326**	68.997
02	68.058	**65.059**	109.479	**98.934**
03	67.022	**65.832**	74.837	**73.197**
04	**47.103**	47.616	99.630	**99.382**
05	67.667	**59.465**	89.686	**87.872**
06	63.709	**62.288**	92.076	**88.542**
07	65.992	**65.442**	83.895	**80.590**
08	73.212	**61.095**	93.830	**93.625**
09	67.226	**64.144**	94.610	**93.393**
10	64.454	**61.207**	83.028	**80.964**

## Data Availability

Not applicable.
